# A Low-Complexity 4D Discrete Chaotic System for Secure Image Encryption Based on Reversible Neural Network

**DOI:** 10.3390/e28070753

**Published:** 2026-07-01

**Authors:** Han Chen, Qingye Huang, Yingjie Su, Lezhu Chen, Baoyi Liao, Linqing Huang, Changwen Chen

**Affiliations:** 1 School of Marine Science and Technology, Shanwei Institute of Technology, Shanwei 516600, China; hanchengdut@163.com (H.C.); lezhuchen@163.com (L.C.); baoyiliao2026@163.com (B.L.); 2 Institute of Collaborative Innovation, University of Macau, Macau 999078, China; 3 College of Information Science and Technology, Jinan University, Guangzhou 510632, China; yingjiesu_jnu@163.com; 4 School of Advanced Manufacturing, Guangdong University of Technology, Jieyang 522000, China; hlq@gdut.edu.cn

**Keywords:** discrete chaotic system, image encryption, reversible neural network, chaotic encryption, security analysis

## Abstract

To address the limitations of existing chaotic systems such as complex structure and potential chaotic degradation, this paper proposes a novel four-dimensional discrete chaotic system (4D-DCS) and an image encryption algorithm based on it. The 4D-DCS is constructed by integrating a feedback controller and modulo operation into a linear discrete-time system, featuring a simple structure without the need for intricate matrix reconstruction or memristor circuits. Mathematical analysis confirms its chaos in the sense of Li–Yorke and numerical simulations including Lyapunov exponent (LE) analysis, 0–1 test, and NIST SP 800-22 test demonstrate its hyperchaotic characteristics and excellent pseudorandomness. Based on the 4D-DCS, the proposed encryption algorithm employs SHA-256 to generate initial states for key uniqueness, combines row–column permutation to disrupt pixel correlation, and adopts a reversible neural network for diffusion to enhance confusion capability. Comprehensive security analysis shows that the algorithm achieves an NPCR of ∼99.61% and a UACI of ∼33.46%, a key space of $2^{216}$, information entropy close to 8, and correlation coefficients of encrypted images near 0. It also exhibits strong robustness against differential, cropping, noise, and chosen-plaintext attacks. Comparative analysis with state-of-the-art algorithms validates the 4D-DCS’s advantages in structural simplicity and stability, and the encryption algorithm’s superiority in security and practicality, making it suitable for security-critical applications such as image encryption.

## 1. Introduction

As core information carriers, images are widely used in security-sensitive fields such as medicine, military, and cloud computing. The need for privacy protection during their transmission and storage is increasingly urgent, requiring image encryption technology to meet the core demands of high security, strong resistance to attacks, and practicality [1].

Chaotic systems have been the mainstream technical cornerstone in the field of image encryption. Low-dimensional chaotic systems exhibit inherent security limitations, such as a narrow key space and vulnerability to cryptographic attacks. By comparison, high-dimensional chaotic systems (4D and above) can enhance resistance against adversarial attacks through increased system complexity, emerging as a prominent research focus in recent years [2,3,4,5,6,7,8,9]. To enhance the complexity of chaotic behavior for applications in chaotic encryption and secure communication, Teng et al. proposed a dual-dynamic-coupling-coefficient coupled map lattice with delayed feedback (DDCMLDF), which introduces two dynamic coupling coefficients and a random delay mechanism based on the traditional coupled map lattice (CML) to achieve a higher degree of chaotic complexity [3]. In 2024, Li et al. constructed a class of chaotic systems that can achieve direct offset boosting for two dimensions via a single constant, featuring multiple typical control modes including system variable single control, synchronous common control, reverse control, and differential control as well as the combination of two-dimensional offset boosting and amplitude control [4]. A novel fractional-order five-dimensional hyper-chaotic system (F5DHS) is proposed by Meng et al. to generate more complicated chaotic sequences for the permutation and diffusion processes in color image encryption [9].

In existing mainstream schemes that combine neural networks with chaotic encryption, the core idea often relies on memristors to simulate the synaptic characteristics of neural networks and the excitation/inhibition behavior between neurons, thereby leveraging the rich dynamic behavior of such memristor neural networks to construct encryption mechanisms [10,11,12,13,14,15,16]. In 2024, Lai et al. proposed an image encryption algorithm with excellent performance by leveraging the multiscroll attractors and coexisting homogeneous/heterogeneous attractors of a novel memristive Hopfield neural network (HNN) [10]. This network is constructed by introducing Sigmoid functions into memristors and has a simple topology with unidirectional neuron connections. To leverage the diverse firing modes and grid multiscroll attractors of the memristive tri-neuron Hopfield neural network (MTN-HNN) constructed by replacing the synapse of the second neuron with a proposed memristor, an encryption scheme was proposed by Yu et al. and successfully implemented on a field-programmable gate array (FPGA) together with custom digital circuits [12]. By leveraging the periodic initial offset boosting behavior and unique multi-scroll attractor extension characteristics of the memristive Hopfield neural network (MHNN)—constructed by coupling a new locally active non-volatile trigonometric memristor into the Hopfield neural network—Leng et al. designed a real-time image encryption scheme for protecting medical image privacy [14].

In parallel, a number of recent works have explored complementary directions for chaos-based image encryption. Nardo et al. exploited the finite-precision error of digital chaotic implementations to construct an encryption scheme with enhanced sensitivity [17]. Nepomuceno et al. proposed a scheme that drives the permutation–diffusion process using the pseudo-orbits of 1D chaotic maps, leveraging the difference between two close pseudo-orbits as the keystream source [18]. Moysis et al. designed a bit-level cubic-shuffling cipher in which image bits are arranged in a three-dimensional matrix and shuffled along its three axes according to chaotic indices [19], while Hua et al. constructed a cosine-transform-based chaotic system that combines two seed chaotic maps via a cosine transform to overcome the chaotic degradation of classical low-dimensional maps [20]. Zhang et al. further developed an image-adaptive encryption algorithm built on a novel 2D enhanced-cosine coupled chaotic system, in which the encryption parameters are adapted to the plaintext to enhance plaintext sensitivity [21].

Nevertheless, existing high-dimensional chaotic systems still suffer from complex and degenerate structures—memristor-based schemes [10,12] rely on intricate circuit design and dedicated hardware, while structure-extended systems [9] involve complex matrix reconstruction or fractional-order calculation that easily causes chaotic degradation in partial parameter ranges. Mainstream neural network-integrated chaotic encryption schemes have the core limitation of deep binding between algorithm design and hardware implementation [10,12,14], requiring deployment on FPGA, DSP platforms or dedicated analog circuits, and their neural network structures are customized for specific chaotic systems with poor scalability and portability. Even the few hardware-independent fusion schemes [11] trade off the structural simplicity of the underlying chaotic system for encryption reversibility, failing to balance system simplicity, encryption security and engineering practicality. This paper aims to construct a high-dimensional chaotic system with low complexity, no degeneracy, and high pseudo-randomness that abandons complex components and hardware dependence, and design a general, hardware-decoupled image encryption algorithm based on this system that achieves an optimal balance of security, computational efficiency and practicality.

The main contributions of this paper are as follows:

1. A novel 4D discrete chaotic system (4D-DCS) with a minimalist structure is proposed, which avoids dependencies on complex components. Through mathematical proofs and numerical simulations, the system exhibits hyperchaotic characteristics and excellent pseudo-randomness, without chaotic degradation.
2. An encryption framework based on 4D-DCS is designed. An initial key bound to the plaintext is generated using the SHA-256 hash function to ensure key uniqueness, row and column scrambling is combined to disrupt pixel spatial correlation, and a reversible neural network is introduced to achieve efficient diffusion, enhance confusion capabilities and support decryption.
3. This encryption algorithm achieves ideal levels in several core security metrics and effectively resists various attacks such as differential attacks, cropping, noise attacks, and chosen-plaintext attacks, making it suitable for security-sensitive scenarios.

The rest of this paper are arranged as follows: Section 2 will elaborate on the design principles, mathematical model, and chaotic characteristic verification process of 4D-DCS. Section 3 will specifically introduce the image encryption algorithm based on this chaotic system, including the three core aspects of key generation, row and column scrambling, and reversible neural network diffusion. Section 4 will verify the comprehensive performance of the algorithm through simulation experiments and multi-dimensional security analysis; Section 5 will summarize the research results of the entire paper and provide an outlook on future optimization directions.

## 2. The Proposed Chaotic System

### 2.1. System Design and Mathematical Model

Current design approaches for chaotic systems typically involve combining existing chaotic systems, tuning their parameters, or splicing their structures. While this strategy enables the rapid development of new systems, it is often confined to the dynamical frameworks of existing chaotic systems, thereby hindering efforts to enhance the system’s complexity and originality at a fundamental level. To address this problem, this paper integrates a feedback controller to transform a linear system into a discrete chaotic system.

For a linear discrete-time system, its state evolution is described as:

(1) $$\begin{array}{c} x_{k+1}=Ax_{k} \end{array}$$

where $A\in R^{m\times n}$ represents the state transition matrix of the linear system. A matrix A is randomly selected such that it satisfies the condition ${∥A∥}_{\infty }<1$.

(2) A=0.7−0.1−0.0−0.00.0−0.6−0.2−0.00.3−0.0−0.5−0.00.0−0.1−0.0−0.8

It is evident that the infinity norm of matrix A is 0.9. A feedback controller $\mu_{k}$ is designed to induce chaotic behavior in this linear discrete-time system, which is shown in Equation (3).

(3) $$\begin{array}{c} \mu_{k}=\left(1+e^{r}\right)x_{k} \end{array}$$

Here, *r* is the control parameter of the system. By integrating the feedback controller $\mu_{k}$ and mod operation into the linear system, the final mathematical model of the novel four-dimensional discrete chaotic system (4D-DCS) is derived as follows:

(4) $$\begin{array}{c} x_{k+1}=\left[A+(1+e^{r})I\right]x_{k}\;\left(mod\;1\right) \end{array}$$

where $x_{k}={\left[x_{k},y_{k},z_{k},w_{k}\right]}^{T}$ and $x_{k+1}={\left[x_{k+1},y_{k+1},z_{k+1},w_{k+1}\right]}^{T}$ denote the 4-dimensional state vectors of the system at discrete time steps *k* and k+1, respectively. The linear expression of the system in terms of state components is further expanded as:

(5) $$\begin{array}{c} \left\{\begin{array}{c} x_{k+1}=\left(1.7+e^{r}\right)x_{k}+0.1y_{k}\;\left(mod\;1\right)\\ y_{k+1}=\left(0.4+e^{r}\right)y_{k}-0.2z_{k}\;\left(mod\;1\right)\\ z_{k+1}=0.3x_{k}+\left(0.5+e^{r}\right)z_{k}\;\left(mod\;1\right)\\ w_{k+1}=0.1y_{k}+\left(1.8+e^{r}\right)w_{k}\;\left(mod\;1\right) \end{array}\right. \end{array}$$

### 2.2. Mathematical Discussion of the 4D-DCS

The original matrix A is already diagonally dominant. Define matrix M as:

(6) $$\begin{array}{c} M=A+(1+e^{r})I \end{array}$$

It is obvious that the matrix M satisfies the diagonally dominant condition:

(7) $$\begin{array}{c} \left|M_{ii}\right|>\sum_{\begin{array}{c} j=1\\ j\neq i \end{array}}^{4}\left|M_{ij}\right| \end{array}$$

According to the non-singularity of diagonally dominant matrices, the inverse matrix $M^{-1}$ of M must exist, and satisfies:

(8) $$\begin{array}{c} M\cdot M^{-1}=I \end{array}$$

Rewrite the equality $M\cdot M^{-1}=I$:

(9) $$\begin{array}{c} \left[(1+e^{r})I+A\right]\cdot M^{-1}=I \end{array}$$

Rearranging terms yields:

(10) $$\begin{array}{c} \left(1+e^{r}\right)I\cdot M^{-1}=I-A\cdot M^{-1} \end{array}$$

Take the infinity norm (${∥\cdot ∥}_{\infty }$) on both sides, and apply the triangle inequality of norms ∥X+Y∥≤∥X∥+∥Y∥:

(11) $$\begin{array}{cc} \left(1+e^{r}\right)\cdot {∥M^{-1}∥}_{\infty }\; & =∥I-AM^{-1}{∥}_{\infty }\\ \; & \leq {∥I∥}_{\infty }+{∥A∥}_{\infty }\cdot {∥M^{-1}∥}_{\infty } \end{array}$$

Rearranging the inequality gives:

(12) $$\begin{array}{c} \left[\left(1+e^{r}\right)-{∥A∥}_{\infty }\right]\cdot {∥M^{-1}∥}_{\infty }\leq 1 \end{array}$$

Given that ${∥A∥}_{\infty }=0.9$, the term $\left(1+e^{r}\right)-{∥A∥}_{\infty }$ is strictly positive. Rearranging the inequality gives:

(13) $$\begin{array}{c} ∥M^{-1}{∥}_{\infty }\leq \frac{1}{\left(1+e^{r}\right)-{∥A∥}_{\infty }}<1 \end{array}$$

Let $b={\left[1,1,1,1\right]}^{T}\in R^{4}$, and it is obvious that ${∥b∥}_{\infty }=1$. Construct the initial state as:

(14) $$\begin{array}{c} x^{0}=M^{-1}\cdot M^{-1}\cdot b \end{array}$$

By the submultiplicativity of norms ∥X·Y∥≤∥X∥·∥Y∥, rearranging the equality:

(15) $$\begin{array}{cc} ∥x^{0}{∥}_{\infty }\; & =∥M^{-1}M^{-1}{b∥}_{\infty }\\ \; & \leq ∥M^{-1}{∥}_{\infty }^{2}\;{∥b∥}_{\infty }\\ \; & <1^{2}\cdot 1=1. \end{array}$$

For the mapping g(x)=Mx(mod1), iterate from the initial state $x^{0}$ to get $x^{1}$ and $x^{2}$:

(16) $$\begin{array}{cc} x^{1} & =g\left(x^{0}\right)=Mx^{0}\;\mathrm{mod}\;1\\ \; & =MM^{-1}M^{-1}b\;\mathrm{mod}\;1\\ \; & =M^{-1}b,\\ x^{2} & =g\left(x^{1}\right)=Mx^{1}\;\mathrm{mod}\;1\\ \; & =MM^{-1}b\;\mathrm{mod}\;1\\ \; & =b\;\mathrm{mod}\;1\\ \; & =0=x^{*}. \end{array}$$

It is obvious that $∥x^{0}∥<∥x^{1}∥<1$. Let *r* denote the radius of the closed ball $B_{r}\left(x^{*}\right)$. Since $∥x^{0}∥<r<∥x^{1}∥$, $x^{0}$ lies inside $B_{r}\left(x^{*}\right)$, while $x^{1}$ is located outside this closed ball.

Let g(x)=Mx(mod1). According to Equation (6), M is a strongly diagonally dominant matrix. By the Gershgorin Disk Theorem, all eigenvalues of M satisfy |λ|>1. Thus, $||Dg(x_{k})||>1$ and $B_{r}\left(x^{*}\right)$ is a repelling domain. In addition, g(x) satisfied:

(17) $$\begin{array}{c} \det\left\{Dg^{2}\left(x^{0}\right)\right\}=\det\left\{Dg\left(x^{0}\right)\right\}\cdot \det\left\{Dg\left(x^{1}\right)\right\}\neq 0 \end{array}$$

So $x^{0}$ is non-degenerate. From the above analysis:

1. There exists a real number r>0 such that for every point $x\in B_{r}\left(x^{*}\right)$, the modulus of all eigenvalues of the Jacobian matrix Dg(x) is greater than 1.
2. There exists a point $x^{0}\in B_{r}\left(x^{*}\right)$ (with $x^{0}\neq x^{*}$) and a natural number m≥2 such that $g^{m}\left(x^{0}\right)=x^{*}$.
3. $x^{0}$ is non-degenerate.

Therefore, $x^{*}=0$ is a recurrent repeller of the 4D-DCS. According to Marotto’s Theorem, the system exhibits chaos in the sense of Li–Yorke.

### 2.3. Bifurcation Diagram and Trajectory

**Figure 1:**
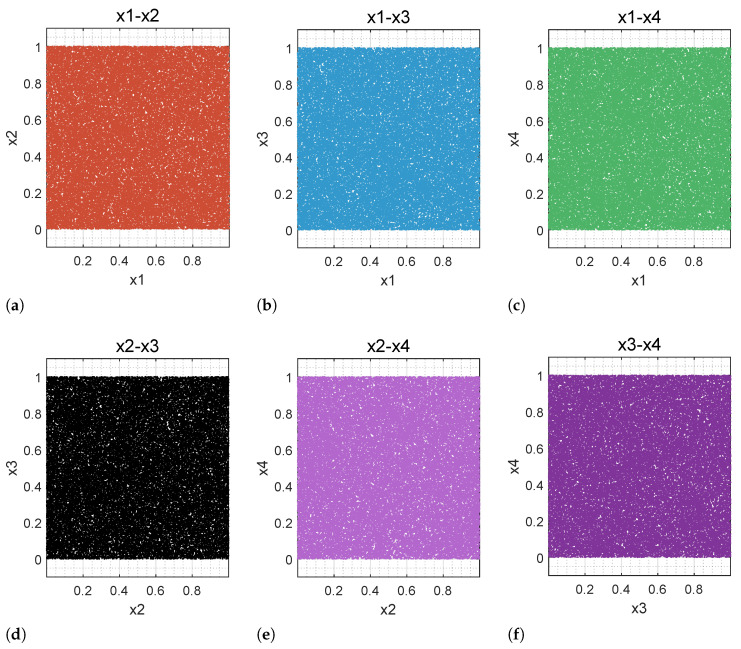
The phase space scatter plot of 4D–DCS. (**a**) x1–x2 plane; (**b**) x1–x3 plane; (**c**) x1–x4 plane; (**d**) x2–x3 plane; (**e**) x2–x4 plane; (**f**) x3–x4 plane.

**Figure 2:**
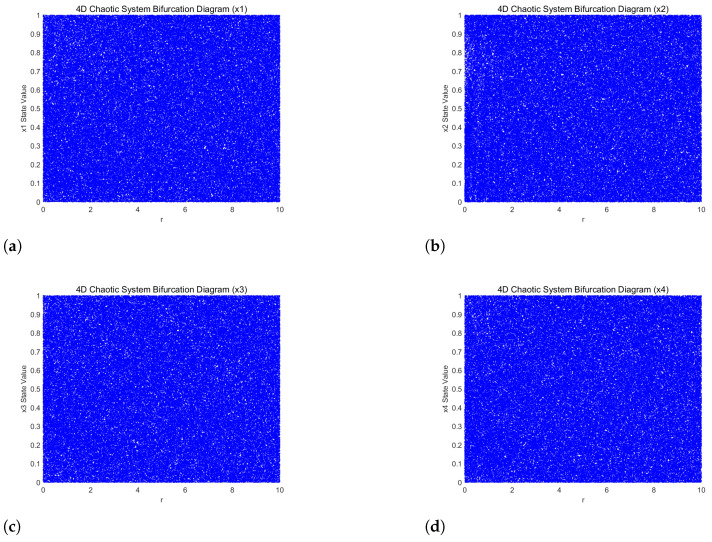
The bifurcation diagram of 4D-DCS for the four state variables. (**a**) x1 plane; (**b**) x2 plane; (**c**) x3 plane; (**d**) x4 plane.

A bifurcation diagram of a chaotic system is a core visualization tool that portrays the long-term dynamical behaviors, enabling intuitive observation of regime transitions. The phase space scatter plot and bifurcation diagram of 4D-DCS is shown in Figure 1 and Figure 2. The phase space scatter plots demonstrate a well-distributed, complex attractor structure across pairwise state variable projections. To avoid relying on purely visual inspection of the bifurcation diagrams, quantitative chaos indicators are computed throughout the same parameter range r∈[0,10]. In Figure 1, the trajectory is generated by iterating Equation (5) for $5\times 10^{4}$ samples with control parameter r=5 and initial state ${\left[0.1,0.2,0.3,0.4\right]}^{T}$; the first $10^{3}$ transient iterations are discarded before plotting. In Figure 2, for each value of the control parameter *r*, the system is iterated for $2\times 10^{3}$ steps and the last $1\times 10^{3}$ samples are recorded. The control parameter *r* is swept over the range [0,10] with a step size of 0.01, yielding 1001 parameter values.

### 2.4. Lyapunov Exponent

The Lyapunov exponent (LE) of a high-dimensional discrete chaotic system can be stably calculated using the QR decomposition method. The core of this method is to separate the perturbation growth factor at each step through the evolution and orthogonal decomposition of the tangent space perturbation, and finally obtain the long-term average exponential separation rate.

For a high-dimensional discrete chaotic system:

(18) $$\begin{array}{c} x_{k+1}=f\left(x_{k}\right) \end{array}$$

where n is the system dimension. f(·) is the non-linear mapping of the system, and $x_{k}$ is the state vector at step k.

Construct the initial perturbation matrix $V_{0}$ (n-order identity matrix).

(19) $$\begin{array}{c} V_{0}=I_{n} \end{array}$$

For each step k=0,1,2,…,N−1: 1. Find the Jacobian matrix $Df(x_{k})$ of the mapping f corresponding to the current state $x_{k}$. 2. Applying the Jacobian matrix to the perturbation matrix $V_{k}$ from the previous step yields a new perturbation matrix:

(20) $$\begin{array}{c} W_{k+1}=Df\left(x_{k}\right)\cdot V_{k} \end{array}$$

3. Perform QR decomposition on $W_{k+1}$ to decompose it into an orthogonal matrix $Q_{k+1}$ and an upper triangular matrix $R_{k+1}$. 4. Update the perturbation matrix for the next iteration to an orthogonal matrix $Q_{k+1}$.

(21) $$\begin{array}{c} V_{k+1}=Q_{k+1} \end{array}$$

5. Record the diagonal elements $r_{ii}^{\left(k+1\right)}$ of the upper triangular matrix $R_{k+1}$ (i=1,2,…,n). After N iterations, for each perturbation direction i, its Lyapunov exponent is the logarithmic mean of the growth factors of all steps:

(22) $$\begin{array}{c} \lambda_{i}=\frac{1}{N}\sum_{k=1}^{N}\ln\left(r_{ii}^{\left(k\right)}\right) \end{array}$$

**Figure 3:**
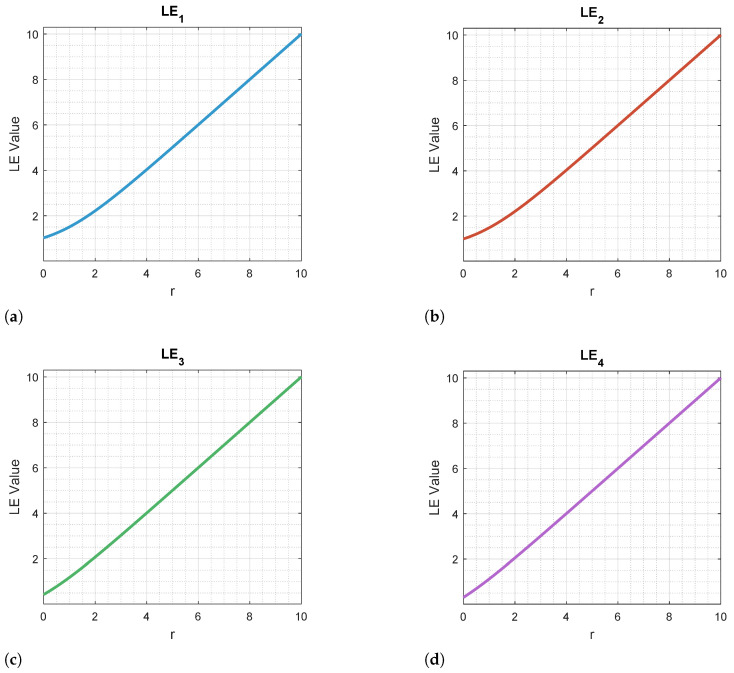
The LE values of 4D-DCS. (**a**) x1–x2 plane; (**b**) x1–x3 plane; (**c**) x1–x4 plane; (**d**) x2–x3 plane.

The LE value of 4D-DCS is shown in Figure 3. One can see that all the LE values are positive, so 4D-DCS is a hyperchaotic system.

### 2.5. Sample Entropy

Sample entropy is a non-linear metric that measures the complexity of a time series data set. The calculation steps are as follows.

Step 1: Given a raw time series {x(i)∣i=1,2,…,N}, extract all consecutive subsequences of length *m* from the raw series to form *m*-dimensional reconstructed vectors.

(23) $$\begin{array}{cc} X_{m}\left(i\right) & ={\left[\;x(i),\;x(i+1),\;\ldots ,\;x(i+m-1)\;\right]}^{\;⊤},\\ i & =1,\;2,\;\ldots ,\;N-m+1. \end{array}$$

Step 2: For each vector $X_{m}\left(i\right)$, compute the Chebyshev distance between $X_{m}\left(i\right)$ and $X_{m}\left(j\right)$ (j≠i, j=1,2,…,N−m+1).

(24) $$\begin{array}{c} d\left(X_{m}\left(i\right),X_{m}\left(j\right)\right)=\max_{1\leq k\leq m}\left|x(i+k-1)-x(j+k-1)\right| \end{array}$$

Step 3: Count the number of vectors $X_{m}\left(j\right)$ satisfying $d\left(X_{m}\left(i\right),X_{m}\left(j\right)\right)\leq r$. For each *i*, calculate the ratio of the matching count to the total number of comparisons (N−m), denoted as $B_{i}$. The average of all $B_{i}$ values is defined as the *m*-dimensional matching probability $B^{m}\left(r\right)$.

(25) $$\begin{array}{c} B^{m}\left(r\right)=\frac{1}{N-m+1}\sum_{i=1}^{N-m+1}\frac{1}{N-m}\times \\ \;\#\;\left\{j\neq i\;|\;d\left(X_{m}\left(i\right),X_{m}\left(j\right)\right)\leq r\right\} \end{array}$$

Step 4: Increase the embedding dimension to m+1 and construct (m+1)-dimensional reconstructed vectors.

(26) $$\begin{array}{cc} X_{m+1}\left(i\right) & ={\left[x(i),x(i+1),\ldots ,x(i+m)\right]}^{T} \end{array}$$

Repeat Step 3 to obtain the average matching probability $A^{m}\left(r\right)$ for (m+1)-dimensional vectors.

SampEn is defined as the negative logarithm of the ratio of $A^{m}\left(r\right)$ to $B^{m}\left(r\right)$:

(27) $$\begin{array}{c} \mathrm{SampEn}\left(m,r,N\right)=-\ln\left(\frac{A^{m}\left(r\right)}{B^{m}\left(r\right)}\right) \end{array}$$

**Figure 4:**
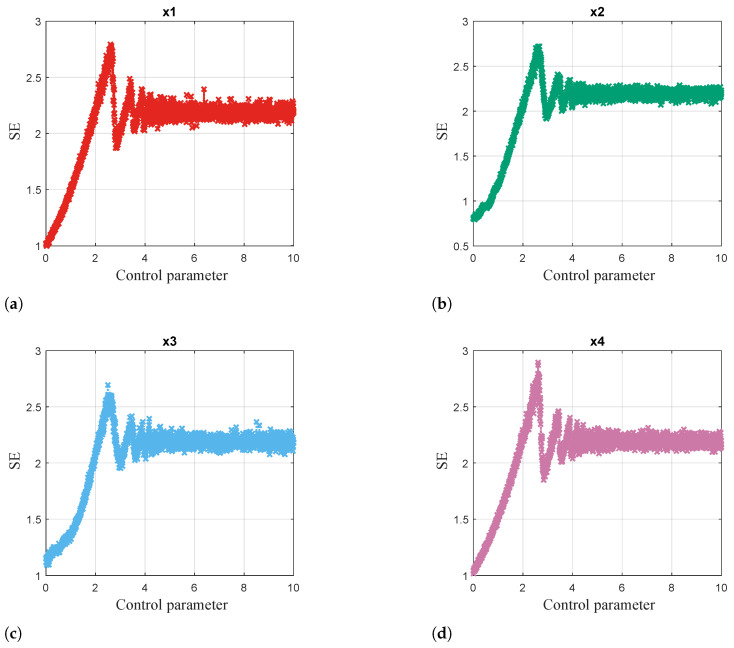
The sample entropy test results of 4D-DCS. (**a**) x1–x2 plane; (**b**) x1–x3 plane; (**c**) x1–x4 plane; (**d**) x2–x3 plane.

In the simulation, the embedding dimension *m* was set to 2, and the similarity tolerance *r* was set to 0.2×std. As shown in Figure 4, the SE values of all variables rise rapidly with the increase of the control parameter and then stabilize at a high level, indicating the excellent pseudo-randomness of the system.

### 2.6. 0–1 Test

The 0–1 test is a widely used method for detecting chaos in deterministic time series. It quantifies chaotic behavior through a binary decision index *K*, where K≈0 suggests regular dynamics, and K≈1 indicates chaotic dynamics. The computation of K proceeds as follows:

Given the discrete time series to be analyzed $S_{j}$ (j=1,2,…,n), construct two cumulative sum sequences $p_{c}\left(n\right)$ and $q_{c}\left(n\right)$ to characterize the cumulative distribution of the original sequence in the trigonometric domain.

(28) $$\begin{array}{c} p_{c}\left(n\right)=\sum_{j=1}^{n}S_{j}\cos\left(jc\right) \end{array}$$

(29) $$\begin{array}{c} q_{c}\left(n\right)=\sum_{j=1}^{n}S_{j}\sin\left(jc\right) \end{array}$$

$p_{c}\left(n\right)$ and $q_{c}\left(n\right)$ correspond to the cumulative cosine and sine components of the sequence, respectively. The translation invariant $M_{c}\left(n\right)$ describes the statistical characteristics of the distance between the sequence and its translated version.

(30) $$\begin{array}{c} M_{c}\left(n\right)=\lim_{N\to \infty }\frac{1}{N}\sum_{j=1}^{n}{\left[p_{c}\left(j+n\right)-p_{c}\left(j\right)\right]}^{2}+{\left[q_{c}\left(j+n\right)-q_{c}\left(j\right)\right]}^{2} \end{array}$$

K is obtained by the logarithmic ratio of the translation invariant $M_{c}\left(n\right)$ to the number of rounds *n*.

(31) $$\begin{array}{c} K=\frac{\log M_{c}\left(n\right)}{\log n} \end{array}$$

**Figure 5:**
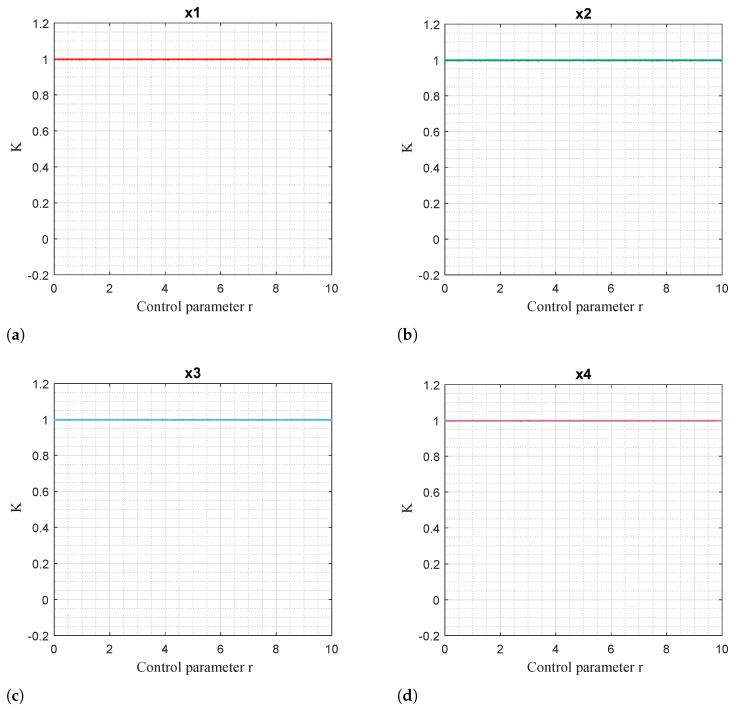
The 0–1 test results of 4D-DCS. (**a**) x1–x2 plane; (**b**) x1–x3 plane; (**c**) x1–x4 plane; (**d**) x2–x3 plane.

The results is shown in Figure 5, all the K values are closed to 1, proving that 4D-DCS achieves high randomness performance.

### 2.7. NIST Test

NIST SP 800-22 is a standard statistical test suite for evaluating the pseudorandomness of bit sequences, commonly applied to verify the quality of chaotic system-generated sequences in cryptographic scenarios. A sequence passes the test if its *p*-value for each sub-test exceeds 0.01, signifying adequate pseudorandomness suitable for secure encryption applications. Each 4D-DCS sequence is iterated 125,000 times. The resulting values are multiplied to $10^{10}$ and then taken modulo 256, generating 1,000,000 bits for the NIST SP 800-22 pseudorandomness test. The NIST test results of 4D-DCS is shown in Table 1. All the *p*-value of 4D-DCS fall in (0.01,1), proving that 4D-DCS is suitable for encryption.

### 2.8. Comparative Analysis

To verify the superiority of the proposed 4D-DCS, a comparative analysis is conducted by benchmarking it against other state-of-the-art 4D chaotic systems. As summarized in Table 2, the proposed 4D-DCS achieves an ultra-high maximal Lyapunov exponent of greater than 10—far exceeding the 2.3020 of Ref. [22] and the sub-1 values of other comparative systems—while maintaining the simplest structural complexity, which make it achieve a superior balance of simplicity, robustness, and practicality.

## 3. The Proposed Encryption Algorithm

**Figure 6:**
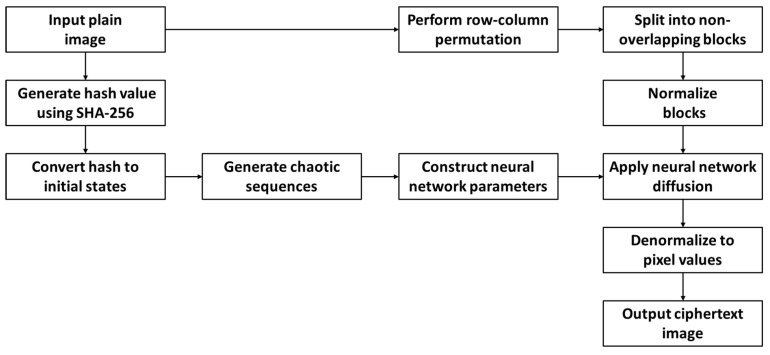
The flowchart of the proposed algorithm.

In this section, all the procedure of the proposed encryption algorithm will be introduced, including key generation, permutation and diffusion operation. The flowchart of the proposed algorithm is shown in Figure 6.

### 3.1. Key Generation

Initially, for the plain image $P\in N^{N\times N}$, the hash value *h* of the image *P* is generated through SHA-256 hash function. The initial values [x,y,z,w] of 4D-DCS will be generated through *h*.

The 64-character hash string is evenly split into four disjoint segments, each containing 16 hexadecimal characters, to ensure balanced randomness across all key elements:

(32) $$\begin{array}{c} S_{1}=h_{1}h_{2}\cdots h_{16} \end{array}$$

(33) $$\begin{array}{c} S_{2}=h_{17}h_{18}\cdots h_{32} \end{array}$$

(34) $$\begin{array}{c} S_{3}=h_{33}h_{34}\cdots h_{48} \end{array}$$

(35) $$\begin{array}{c} S_{4}=h_{49}h_{50}\cdots h_{64} \end{array}$$

Each segment $S_{k}$ (k=1,2,3,4) corresponds to a 64-bit binary value, and then is converted to a 64-bit decimal integer:

(36) $$\begin{array}{c} D_{k}=\sum_{i=1}^{16}\mathrm{val}\left(s_{k,i}\right)\cdot 16^{16-i} \end{array}$$

where $\mathrm{val}\left(\cdot \right):H\to N_{0}$ is the hexadecimal-to-numeric mapping function, defined as:

(37) $$\begin{array}{c} \mathrm{val}\left(s\right)=\left\{\begin{array}{cc} s, & s\in \{0,1,\ldots ,9\}\\ 10+(s-a), & s\in \{a,b,\ldots ,f\} \end{array}\right. \end{array}$$

To align with the stable initial state range [0.01,1.0] of the chaotic system, each decimal integer $D_{k}$ is linearly mapped to the target interval via the affine transformation:

(38) $$\begin{array}{c} k_{k}=0.01+\left(1.0-0.01\right)\cdot \frac{D_{k}}{2^{64}-1} \end{array}$$

This transformation guarantees x,y,z,w∈[0.01,1.0], which is critical for avoiding chaotic system divergence. Finally, using the mapped initial values x,y,z,w as the initial states, four independent chaotic sequences $\chi_{x},\chi_{y},\chi_{z},\chi_{w}$ are generated via iterative evolution of Equation (5).

### 3.2. Permutation Operation

To generate row and column permutation indices, $\chi_{x}$ is split into two disjoint subsequences:

(39) $$\begin{array}{c} \chi_{\mathrm{row}}=\left[\chi_{x,1},\chi_{x,2},\ldots ,\chi_{x,M}\right]\in R^{M} \end{array}$$

(40) $$\begin{array}{c} \chi_{\mathrm{col}}=\left[\chi_{x,M+1},\chi_{x,M+2},\ldots ,\chi_{x,M+N}\right]\in R^{N} \end{array}$$

Each element of $\chi_{\mathrm{row}}$ and $\chi_{\mathrm{col}}$ is then transformed to an integer within the valid index range via modular arithmetic:

(41) $$\begin{array}{cc} {\tilde{\chi }}_{\mathrm{row},i} & =\mathrm{mod}\left(\left\lfloor \chi_{\mathrm{row},i}\times 10^{15}\right\rfloor ,M\right),\;i=1,2,\ldots ,M \end{array}$$

(42) $$\begin{array}{cc} {\tilde{\chi }}_{\mathrm{col},j} & =\mathrm{mod}\left(\left\lfloor \chi_{\mathrm{col},j}\times 10^{15}\right\rfloor ,N\right),\;j=1,2,\ldots ,N \end{array}$$

Permutation indices are derived by sorting the transformed chaotic subsequences and extracting the sorted indices.

Let $S:R^{K}\to N^{K}$ denote the sorting operator. The row and column permutation indices are defined as:

(43) $$\begin{array}{c} \left\{\begin{array}{c} r=S\left({\tilde{\chi }}_{\mathrm{row}}\right)=\left[r_{1},r_{2},\ldots ,r_{M}\right]\in N^{M},\\ c=S\left({\tilde{\chi }}_{\mathrm{col}}\right)=\left[c_{1},c_{2},\ldots ,c_{N}\right]\in N^{N}, \end{array}\right. \end{array}$$

where $r_{i}\in \left\{1,2,\ldots ,M\right\}$ and $c_{j}\in \left\{1,2,\ldots ,N\right\}$.

The permuted image $P_{\mathrm{perm}}\in Z_{256}^{M\times N}$ is constructed by reordering the rows and columns of P using r and c.

(44) $$\begin{array}{c} P_{\mathrm{perm}}\left(i,j\right)=P\left(r_{i},c_{j}\right),\;\forall i\in \left\{1,\ldots ,M\right\},j\in \left\{1,\ldots ,N\right\} \end{array}$$

Here, P(i,j) denotes the pixel value of *P* at row *i* and column *j*. The permutation is invertible, as r and c can be used to reverse the row/column reordering during decryption.

### 3.3. Diffusion Operation with a Reversible Neural Network

Compared with conventional element-wise XOR or modular-addition diffusion, employing a reversible neural network (RNN) in the diffusion stage offers three concrete advantages. (i) Strictinvertibility without lookup tables. The linear layer is parameterized by an orthogonal matrix *W* ($W^{T}W=I$) generated from the chaotic sequence $\chi_{y}$, so its inverse equals its transpose and decryption is bit-exact, avoiding the rounding errors that often plague non-orthogonal neural ciphers. (ii) Stronger confusion through non-linear coupling. The hyperbolic-tangent activation, combined with the chaotic bias *b* derived from $\chi_{z}$, induces a highly non-linear input–output mapping at the block level, which propagates a single-bit plaintext change across the entire 16×16 block in one round and thereby boosts plaintext sensitivity (NPCR/UACI). (iii) Block-parallel computational efficiency. The diffusion is performed in $T_{b}$ independent 16×16 blocks via dense matrix multiplications, which can be evaluated in parallel and exhibit a per-pixel cost of O(d) (with d=256) that is asymptotically comparable to a single XOR sweep but with substantially higher diffusion capability per round.

The image *P* is split into $T_{b}$ non-overlapping 16×16 blocks. This yields a block matrix:

(45) $$\begin{array}{c} F={\left[f_{1},f_{2},\ldots ,f_{T_{b}}\right]}^{T}\in R^{T_{b}\times d}, \end{array}$$

where $f_{k}\in R^{1\times d}$ is the flattened *k*-th block.

Each block is normalized to [−1,1] with block-wise minima/maxima stored for decryption:

(46) $$\begin{array}{c} m_{k}=\min\left(f_{k}\right),\;M_{k}=\max\left(f_{k}\right)\;\left(k=1,\ldots ,T_{b}\right), \end{array}$$

(47) $$\begin{array}{c} F_{\mathrm{norm}}\left[k,:\right]=2\cdot \frac{f_{k}-m_{k}}{M_{k}-m_{k}+\epsilon }-1, \end{array}$$

where $m={\left[m_{1},m_{2},\ldots ,m_{T_{b}}\right]}^{T}$ (block minima) and $M={\left[M_{1},M_{2},\ldots ,M_{T_{b}}\right]}^{T}$ (block maxima) are saved for inversion.

An orthogonal matrix $W\in R^{d\times d}$ is constructed from the chaotic sequence $\chi_{y}$.

1. Extract a subsequence from $\chi_{y}$: $\chi_{y}^{W}=\chi_{y}\left[1:d^{2}\right]\in R^{d^{2}}$.
2. Min–max normalization to [0,1]:
  (48) $$\begin{array}{c} {\hat{\chi }}_{y}^{W}=\frac{\chi_{y}^{W}-\min\left(\chi_{y}^{W}\right)}{\max\left(\chi_{y}^{W}\right)-\min\left(\chi_{y}^{W}\right)+\epsilon }, \end{array}$$
  where $\epsilon =10^{-16}$.
3. Rescale to [−1,1]:
  (49) $$\begin{array}{c} {\tilde{\chi }}_{y}^{W}=2{\hat{\chi }}_{y}^{W}-1 \end{array}$$
4. Reshape to a square matrix (d=16):
  (50) $$\begin{array}{c} C_{W}=\mathrm{reshape}\left({\tilde{\chi }}_{y}^{W},d,d\right)\in R^{d\times d} \end{array}$$
5. QR decomposition to obtain an orthogonal matrix:
  (51) $$\begin{array}{c} C_{W}=QR, \end{array}$$
  where $Q\in R^{d\times d}$ is orthogonal and *R* is upper triangular.
6. Scale *Q* with a chaotic mean-based factor:
  (52) $$\begin{array}{c} \mu_{W}=\frac{1}{d^{2}}\sum_{i=1}^{d^{2}}\chi_{y}^{W}\left[i\right] \end{array}$$
  (53) $$\begin{array}{c} \sigma_{W}=\alpha \cdot (1+|\mu_{W}\left|\right) \end{array}$$
  (54) $$\begin{array}{c} W=Q\cdot \sigma_{W} \end{array}$$

The $b\in R^{d\times 1}$ is derived from the chaotic sequence $\chi_{z}$ to introduce non-linear offset:

1. Extract a subsequence from $\chi_{z}$:
  (55) $$\begin{array}{c} \chi_{z}^{b}=\chi_{z}\left[1:d\right]\in R^{d} \end{array}$$
2. Min–max normalization to [0,1]:
  (56) $$\begin{array}{c} {\hat{\chi }}_{z}^{b}=\frac{\chi_{z}^{b}-\min\left(\chi_{z}^{b}\right)}{\max\left(\chi_{z}^{b}\right)-\min\left(\chi_{z}^{b}\right)+\epsilon }. \end{array}$$
3. Rescale to [−1,1]:
  (57) $$\begin{array}{c} {\tilde{\chi }}_{z}^{b}=2{\hat{\chi }}_{z}^{b}-1 \end{array}$$
4. Scale with α and reshape to a column vector:
  (58) $$\begin{array}{c} b=\mathrm{reshape}({\tilde{\chi }}_{z}^{b}\cdot \alpha ,d,1). \end{array}$$

The normalized blocks are transformed via the chaotic neural layer:

(59) $$\begin{array}{c} L=F_{\mathrm{norm}}\cdot W^{T}+b^{T}\in R^{T_{b}\times d}, \end{array}$$

A hyperbolic tangent activation (tanh) is applied to introduce non-linearity:

(60) $$\begin{array}{c} A=\tanh\left(L\right)\in R^{T_{b}\times d}, \end{array}$$

The activated blocks are denormalized back to 8-bit pixel values:

(61) $$\begin{array}{c} F_{\mathrm{enc}}\left[k,:\right]=\mathrm{uint}8\left((A_{\mathrm{clip}}\left[k,:\right]+1)\cdot 127.5\right), \end{array}$$

where 127.5=255/2 (scales [−1,1] to [0,255]). The encrypted block matrix $F_{\mathrm{enc}}$ is reshaped back to the original image dimensions:

(62) $$\begin{array}{c} P_{\mathrm{enc}}=\mathrm{reshape}\left(\mathrm{unvectorize}\left(F_{\mathrm{enc}}\right),M,N\right)\in Z_{256}^{M\times N} \end{array}$$

where unvectorize(·) reverses the block flattening operation. Finally, performed XOR operation between the $P_{\mathrm{enc}}$ and the chaotic sequence $\chi_{w}$.

Decryption is the reverse process of encryption. First, perform an inverse XOR operation between the ciphertext image and the chaotic sequence $\chi_{w}$, then use the stored block extrema for denormalization reversal, apply the $\tanh^{-1}$ inverse activation and the transpose of the orthogonal matrix *W* for reverse linear transformation, and recover and reorganize the permuted image blocks. Finally, execute inverse permutation using the row and column permutation indices stored during encryption.

## 4. Simulation and Security Analysis

The experimental setup was configured with a laptop equipped with an AMD Ryzen 9 7940HX processor and 16.0 GB of RAM, while MATLAB R2022a was adopted as the dedicated simulation platform.

### 4.1. PSNR and SSIM Analysis

Peak Signal-to-Noise Ratio (PSNR) and Structural Similarity Index Measure (SSIM) are two widely adopted metrics for evaluating image quality: the former quantifies pixel-level discrepancies between two images, while the latter assesses the structural consistency of image content—both serve as effective indicators for analyzing image encryption performance. For grayscale images, the PSNR value is derived from the Mean Squared Error (MSE) of the original image (P) and the target image (C), calculated via Equation (63):

(63) $$\left\{\begin{array}{cc} \mathrm{PSNR} & =10\mathrm{lg}\left(\frac{255^{2}}{\mathrm{MSE}}\right)\\ \mathrm{MSE} & =\frac{\sum_{i=1}^{M}\sum_{j=1}^{N}{\left(P(i,j)-C(i,j)\right)}^{2}}{M\times N} \end{array}\right.$$

Here, M and N denote the width and height of the two images. A smaller PSNR value corresponds to a more significant pixel-level gap between the images. In contrast, SSIM evaluates image similarity by integrating luminance, contrast and structural components, with its calculation for 8-bit grayscale images given in Equation (64):

(64) $$\begin{array}{c} \mathrm{SSIM}\left(P,C\right)=\frac{\left(2\mu_{P}\mu_{C}+C_{1}\right)\left(2\sigma_{PC}+C_{2}\right)}{\left(\mu_{P}^{2}+\mu_{C}^{2}+C_{1}\right)\left(\sigma_{P}^{2}+\sigma_{C}^{2}+C_{2}\right)} \end{array}$$

where $\mu_{P}/\mu_{C}$ are the means of P/C, $\sigma_{P}^{2}/\sigma_{C}^{2}$ are their variances, $\sigma_{PC}$ is their covariance, and $C_{1}={\left(K_{1}\times 255\right)}^{2}$, $C_{2}={\left(K_{2}\times 255\right)}^{2}$ ($K_{1}=0.01$, $K_{2}=0.03$) are stability constants.

A SSIM value closer to 0 indicates negligible structural overlap between the two images. The PSNR and SSIM values of the proposed method are presented in Table 3.

### 4.2. Differential Attacks

To assess the sensitivity of encryption algorithms to plaintext modifications, the number of pixel change rate (NPCR) and unified average changing intensity (UACI) are commonly employed. The computational formulas for these two evaluation metrics are formally defined in Equation (65).

(65) $$\left\{\begin{array}{cc} NPCR & =\sum_{i=0}^{H}\sum_{j=0}^{W}D\left(i,j\right)\times 100\%,\\ UACI & =\frac{1}{W\times H}\sum_{i=0}^{H}\sum_{j=0}^{W}\frac{\left|c_{1}\left(i,j\right)-c_{2}\left(i,j\right)\right|}{255}\times 100\%. \end{array}\right.$$

Here, W and H represent the width and height of the two images. The binary indicator function D(i,j) is defined as follows: D(i,j)=0 if the pixels $c_{1}\left(i,j\right)$ and $c_{2}\left(i,j\right)$ are identical and D(i,j)=1 if they are different. For an encryption algorithm to exhibit excellent plaintext sensitivity, it should achieve a high NPCR value (surpassing 99.6094%) and an appropriate UACI value (approximately 33.4635%). As presented in Table 4, all the calculated NPCR and UACI values of the proposed model are closely aligned with their respective ideal benchmarks. This result verifies that the designed encryption scheme can effectively withstand differential attacks.

### 4.3. Exhaustive Attack Analysis

A secure encryption algorithm should be equipped with a key space of no less than $2^{100}$ to fend off potential adversarial attacks. The proposed encryption framework adopts a multi-parameter key set, where $key_{i}\in \left(0,4\right)$ for i=1,2,3,4, and r∈(0,150). With a computational precision set to $10^{-15}$, the total key space of the system is derived as shown in Equation (66):

(66) $$keyspace=150\times 1800\times {\left(10^{15}\right)}^{4}\approx 2^{216}\gg 2^{100}$$

Since the calculated key space of $2^{216}$ far exceeds the recommended minimum threshold of $2^{100}$, this result fully verifies that the proposed encryption algorithm exhibits robust resistance against brute-force attacks.

### 4.4. Statistical Attack Analysis

#### 4.4.1. Histogram Analysis

**Figure 7:**
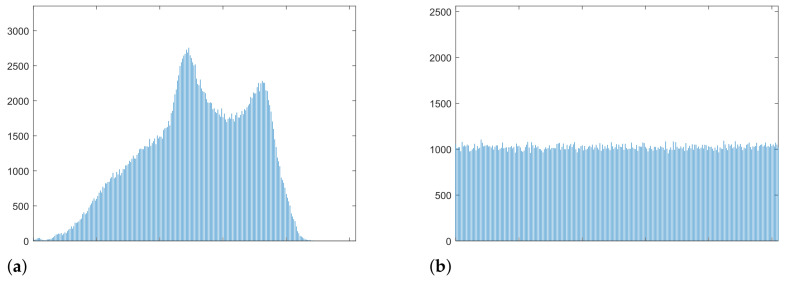
Histogram test results using Baboon image. (**a**): Original image histogram. (**b**): Encrypted image histogram.

Histogram analysis serves to assess the randomness of ciphertext images by examining the distribution of their gray-scale values. Specifically, if an encryption algorithm can achieve uniform distribution of the plaintext image’s gray-scale values—resulting in a nearly flat histogram for the encrypted image—adversaries will find it difficult to extract actionable information about the original image through statistical methods. This, in turn, strengthens the algorithm’s resilience against statistical attacks. As illustrated in Figure 7, the histogram of the encrypted image exhibits a flat distribution, which means attackers are unlikely to glean meaningful clues about the plaintext via statistical analysis.

#### 4.4.2. Correlation Coefficient Test

The correlation coefficient test is primarily used to evaluate the randomness and security performance of ciphertext images by analyzing the correlation between adjacent pixels, with the specific calculation method given in Equation (67).

(67) $$\left\{\begin{array}{c} r_{xy}=\frac{\mathrm{cov}(x,y)}{\sqrt{D(x)}\times \sqrt{D(y)}}\\ cov\left(x,y\right)=\frac{1}{N}\sum_{i=0}^{N}\left(x_{i}-E\left(x\right)\right)\left(y_{i}-E\left(y\right)\right)\\ D\left(x\right)=\frac{1}{N}\sum_{i=0}^{N}{\left(x_{i}-E\left(x\right)\right)}^{2}\\ E\left(x\right)=\frac{1}{N}\sum_{i=0}^{N}x_{i} \end{array}\right.$$

**Figure 8:**
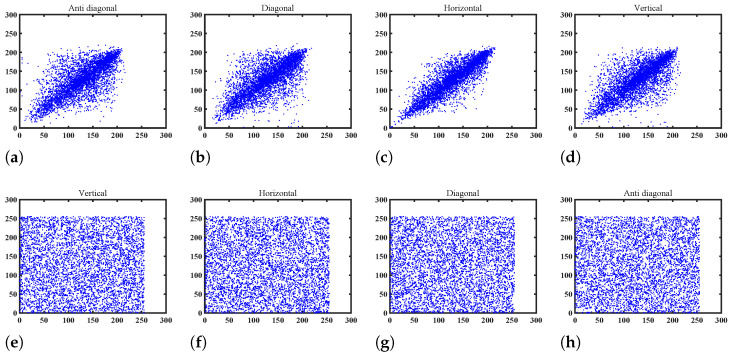
Correlation test results using image Baboon. (**a**–**d**): The correlation test results of the original image in four directions. (**e**–**h**): The correlation test results of the encrypted image in four directions.

Adjacent pixels in plaintext images typically exhibit strong correlation; in contrast, a secure encryption algorithm should markedly diminish this correlation after encryption. This is to prevent attackers from exploiting pixel correlation to recover the original image information. In this simulation, 5000 pairs of adjacent pixels were randomly selected from both the original natural image and its corresponding ciphertext image. The detailed results of the correlation coefficient analysis are presented in Table 5 and visualized in Figure 8. As shown in these results, the correlation coefficients of all ciphertext images are close to 0, demonstrating that there is no significant correlation between adjacent pixels in the encrypted images.

#### 4.4.3. Information Entropy Analysis

Information entropy H(m) serves as a core metric to assess the uncertainty or randomness of information, quantifying both the average information content and the level of uncertainty associated with a random variable. The specific computational formula for H(m) is given in Equation (68).

(68) $$H\left(m\right)=\sum_{i=0}^{N}p\left(m_{i}\right)\log\frac{1}{p(m_{i})}$$

Here, $p(m_{i})$ denotes the probability that the random variable *m* assumes the value $m_{i}$, while *N* represents the total number of all possible values of *m*. For an 8-bit grayscale image, each pixel’s grayscale value can take an integer between 0 and 255, corresponding to 256 possible values in total. If the image’s grayscale value distribution is perfectly uniform, its information entropy will attain the maximum value of 8. As shown in Table 6, the calculated H(m) values of the encrypted images are close to 8, which demonstrates that the grayscale value distribution of the encrypted images is highly uniform. This makes it challenging for attackers to extract actionable information about the plaintext through statistical analysis.

### 4.5. Chosen-Plaintext and Chosen-Ciphertext Attacks

#### 4.5.1. Chosen-Plaintext Attack (CPA)

**Figure 9:**
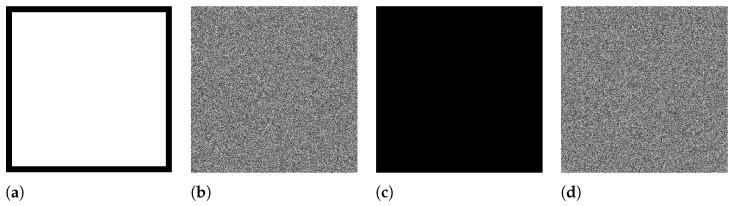
Chosen-plaintextattack test results. (**a**) The all-white figure; **(b**) The encryption result of (**a**); (**c**) The all-black figure; (**d**) The encryption result of (**c**).

The chosen-plaintext attack represents a typical category of cryptanalytic approaches. In this attack scenario, adversaries are allowed to arbitrarily select specific plaintexts and acquire their corresponding ciphertexts. By dissecting the inherent correlations between the plaintexts and ciphertexts, attackers strive to deduce the secret key of the encryption algorithm or unravel its internal operational structure. As a general criterion for security evaluation, if a cryptographic system is capable of generating ciphertext images with a noise-like appearance when encrypting extreme plaintext images (such as all-black or all-white ones), this is a clear indication of its strong robustness against chosen-plaintext attacks. As illustrated in Figure 9, the ciphertext images of both all-white and all-black plaintexts exhibit a uniformly noise-like appearance with no perceptible structure, confirming that the proposed algorithm offers strong resistance against chosen-plaintext attacks.

#### 4.5.2. Chosen-Ciphertext Attack (CCA)

In a chosen-ciphertext attack, the adversary is granted access to a decryption oracle and can query the decryption of arbitrarily chosen ciphertexts (except the target one) in order to recover the secret key or the original plaintext. The proposed scheme resists CCA through three coupled mechanisms. The initial states [x,y,z,w] of the 4D-DCS are derived from the SHA-256 hash of the plaintext itself, so each plaintext induces an essentially distinct key stream; a ciphertext obtained for one plaintext provides no useful information about the key stream associated with another plaintext. Flipping a single bit of the key changes the resulting ciphertext almost completely: under a 1-bit perturbation of the initial state, the measured NPCR and UACI between the two ciphertexts of the Baboon image are 99.6093% and 33.4612%, respectively, both of which are essentially at the ideal benchmarks. Although the RNN diffusion layer is invertible with the correct *W* and *b*, both quantities are generated from the chaotic sequences $\chi_{y}$ and $\chi_{z}$ that are protected by the plaintext-bound key. Without exact knowledge of these chaotic sequences, applying the formal inverse to a queried ciphertext yields a pseudo-random image that exposes no plaintext structure. Consequently, the proposed scheme provides practical CCA resistance in addition to the CPA resistance demonstrated above.

### 4.6. Cropping and Noise Attack

**Figure 10:**
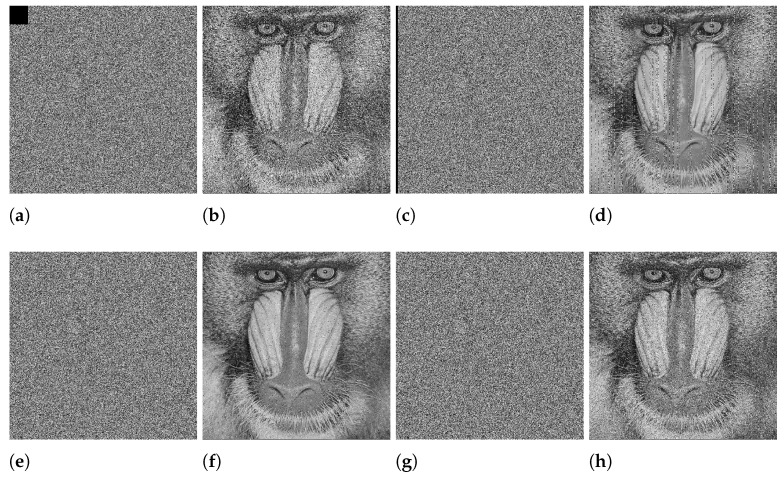
Croppingand noise attack results using Baboon image. (**a**) 1% cropping attack; (**b**) Decryption image of (**a**); (**c**) 1% cropping attack; (**d**) Decryption image of (**c**); (**e**) 0.1% salt and pepper noise attack; (**f**) Decryption image of (**e**); (**g**) 0.5% salt and pepper noise attack; (**h**) Decryption image of (**g**).

When encrypted data is transmitted over public communication channels, data integrity may be impaired by noise interference or partial data loss. As presented in Figure 10 and Table 7, the proposed encryption algorithm exhibits remarkable robustness under these adverse scenarios. Notably, even when the ciphertext image suffers from noise corruption or partial data cropping, the algorithm still retains the ability to recover the core information of the original plaintext image.

### 4.7. Computational Complexity Analysis

The asymptotic time complexity of the proposed scheme is analyzed stage by stage for a grayscale image of size M×N with $T_{b}=MN/d$ non-overlapping blocks of dimension d=256.

- Keygeneration. SHA-256 hashing of the plaintext is O(MN), and the subsequent fixed-length affine mapping is O(1).
- Chaotic sequence generation. Iterating the 4D-DCS (Equation (5)) to produce a sequence of length *L* costs O(L), where $L=\max(M+N,\;d^{2})$ is dominated by the diffusion layer size.
- Row–column permutation. Sorting two chaotic subsequences of lengths *M* and *N* requires O(MlogM+NlogN), and reordering pixels is O(MN).
- Reversible-neural-network diffusion. Constructing the orthogonal weight matrix *W* via QR decomposition is $O(d^{3})$ and is computed only once; applying *W* to all $T_{b}$ blocks costs $O\left(T_{b}\cdot d^{2}\right)=O\left(MN\cdot d\right)$, and the element-wise tanh activation and final XOR add O(MN).

Combining all stages, the overall complexity is

(69) $$\begin{array}{c} T\left(M,N\right)=O\left(MN\cdot d\right)+O\left(d^{3}\right), \end{array}$$

which is linear in the number of pixels for any fixed block dimension *d*. The dominant constant factor stems from the dense linear layer, but it is offset by the block-parallel structure: the $T_{b}$ blocks are mutually independent and can be processed concurrently on multi-core CPUs or GPUs. The decryption procedure performs the same operations in reverse order and shares the identical complexity bound.

### 4.8. Comparative Analysis

To validate the security and performance superiority of the proposed encryption algorithm, a comparative analysis is performed against ten state-of-the-art image encryption methods by evaluating core security metrics including plaintext sensitivity (NPCR/UACI), key space, entropy, and correlation coefficient.

As shown in Table 8, the proposed method achieves an NPCR of 99.6089% and a UACI of 33.4599%, which are highly consistent with the ideal benchmarks for resisting differential attacks. While some algorithms [27] have a larger key space, their key generation relies on complex matrix reconstruction and fractional-order calculation, leading to an increase in computational cost of chaotic sequence generation; in contrast, the proposed method’s key space of $2^{216}$ far exceeds the secure threshold of $2^{100}$, and its key generation is based on SHA-256 and simple chaotic iteration, achieving a balance between key space size and generation efficiency. The proposed method’s information entropy of 7.9993 is close to the theoretical maximum of 8, and its correlation coefficients in three directions are all near 0, which is comparable to the best-performing algorithms [27,28] in resisting statistical attacks.

## 5. Conclusions

This paper proposes a novel four-dimensional discrete chaotic system 4D-DCS constructed by integrating a feedback controller and modulo operation into a linear system, which features simple structure, no chaotic degradation, and excellent pseudorandomness verified by Lyapunov exponent, 0–1 test, and NIST SP 800-22 test. Based on this system, an image encryption algorithm is designed with SHA-256-based key generation, row–column permutation, and reversible neural network-based diffusion. Comprehensive security analysis shows the algorithm achieves ideal NPCR/UACI values, large key space, high information entropy, and strong resistance to differential, statistical, cropping, and noise attacks, outperforming many state-of-the-art methods in terms of balance between security and practicality. Despite the favorable security and complexity properties demonstrated above, the present study has several limitations that should be acknowledged. First, the experimental validation is restricted to 8-bit grayscale natural images; extensions to color images, medical volumetric data, and video streams remain to be empirically verified. Second, the proposed scheme is evaluated purely at the algorithm level on a general-purpose CPU; a dedicated hardware realization that would quantify on-chip throughput, energy efficiency, and side-channel resistance has not yet been carried out. Future work will focus on optimizing the neural network structure and algorithm workflow to improve encryption speed, extending the scheme to video encryption, realizing the proposed system on FPGA/ASIC and memristive crossbar platforms to evaluate its hardware throughput and energy efficiency, and exploring the integration of quantum computing or lightweight cryptographic technologies to enhance its adaptability in more security-critical application scenarios.

## Figures and Tables

**Table 1:** NIST SP 800-22 test results of 4D-DCS.

TEST	*p*-Value of X	*p*-Value of Y	*p*-Value of Z	*p*-Value of W	Result
Frequency	0.181557	0.366918	0.657933	0.085587	PASS
BlockFrequency	0.911413	0.494392	0.798139	0.042808	PASS
CumulativeSums	0.598515	0.447004	0.466572	0.485031	PASS
Runs	0.262249	0.419021	0.983453	0.867692	PASS
LongestRun	0.494392	0.616305	0.213309	0.419021	PASS
Rank	0.637119	0.851383	0.455937	0.401199	PASS
FFT	0.554420	0.554420	0.657933	0.637119	PASS
NonOverlappingTemplate	0.509773	0.476034	0.474789	0.503302	PASS
Universal	0.637119	0.678686	0.759756	0.554420	PASS
OverlappingTemplate	0.514124	0.595549	0.955835	0.304126	PASS
ApproximateEntropy	0.595549	0.071177	0.455937	0.798139	PASS
RandomExcursions	0.493004	0.385492	0.333062	0.173022	PASS
RandomExcursionsVariant	0.581827	0.449237	0.307592	0.442539	PASS
Serial	0.370754	0.350485	0.590983	0.441377	PASS
LinearComplexity	0.455937	0.162606	0.383827	0.437274	PASS

**Table 2:** Comparison of core experimental results of 4D chaotic systems.

Work	Dimension	Maximal LE Value	Hyperchaotic	Degenerate
Ref. [22]	4D	2.3020	Yes	No
Ref. [23]	4D	0.0032	No	No
Ref. [24]	4D	0.8950	No	No
Ref. [25]	4D	0.6500	No	No
Ref. [26]	4D	0.3824	No	No
Proposed	4D	>10	Yes	No

**Table 3:** PSNR and SSIM values of original and decryption images.

Image	Baboon	Airfield	Flower	Fruits	Sailboat
PSNR	41.7541	39.5760	41.4498	41.3397	40.6352
SSIM	0.9941	0.9876	0.9861	0.9860	0.9876

**Table 4:** NPCR and UACI values of original and decryption images.

Image	Baboon	Airfield	Flower	Fruits	Sailboat
NPCR	99.6114	99.6099	99.6087	99.6101	99.6046
UACI	33.4638	33.4441	33.4655	33.4666	33.4597

**Table 5:** The correlation analysis of original images and encrypted images.

Image		Vertical	Horizontal	Diagonal
Baboon	Original	0.7463	0.8704	0.7260
	Encrypted	0.0157	−0.0171	0.0131
Airfield	Original	0.9401	0.9432	0.8995
	Encrypted	−0.0146	−0.0124	−0.0018
Flower	Original	0.9887	0.9919	0.9798
	Encrypted	0.0004	−0.0226	−0.0136
Fruits	Original	0.9853	0.9851	0.9696
	Encrypted	−0.0025	0.0055	−0.0092
Sailboat	Original	0.9687	0.9741	0.9568
	Encrypted	−0.0342	−0.0073	0.0260
Cameraman	Original	0.9557	0.9207	0.9078
	Encrypted	0.0092	0.0116	0.0075

**Table 6:** The information entropy value for various images.

Image	Baboon	Fruits	Flower	Airfield	Sailboat
Original	7.3585	7.3644	7.4107	7.1206	7.4853
Encrypted	7.9994	7.9993	7.9992	7.9993	7.9992

**Table 7:** PSNR and SSIM values of original and decryption images of cropping and noise attack.

Image	Figure 10b	Figure 10d	Figure 10f	Figure 10h
PSNR	11.8274	15.5731	21.0404	14.6781
SSIM	0.2144	0.5392	0.6982	0.3415

**Table 8:** Comparativeanalysis of the proposed method with state-of-the-art algorithms.

Algorithm	Plaintext Sensitivity		Key Space	Entropy	Correlation Coefficient (CC)		
	NPCR (%)	UACI (%)			Vertical	Horizontal	Diagonal
Ref. [29]	99.6094	33.4459	$2^{512}$	7.9973	0.0069	−0.0011	−0.0007
Ref. [30]	99.6451	33.4938	$2^{832}$	7.9991	0.0051	0.0024	0.0063
Ref. [31]	100.0000	33.3700	$2^{256}$	7.9990	0.0068	−0.0030	0.0081
Ref. [27]	99.6521	33.4721	$2^{1000}$	7.9997	0.0002	0.0021	0.0002
Ref. [28]	99.6124	33.4569	$2^{797}$	7.9995	−0.0001	0.0002	−0.0003
Ref. [32]	99.6600	28.3400	$2^{128}$	7.9972	0.0010	−0.0037	−0.0029
Ref. [33]	99.6085	33.4620	$2^{475}$	7.9971	0.0052	−0.0008	−0.0012
Ref. [34]	99.6154	33.5369	$2^{100}$	7.9973	−0.0064	−0.0111	0.0003
Ref. [35]	99.6082	33.4635	$2^{512}$	7.9993	−0.0019	0.0008	−0.0013
Ref. [36]	99.6000	33.4000	$2^{449}$	7.9900	−0.0003	−0.0003	−0.0003
Proposed Method	99.6089	33.4599	$2^{216}$	7.9993	−0.0043	−0.0071	0.0037

## Data Availability

The datasets generated and/or analyzed during the current study are available from the https://www.hlevkin.com/hlevkin/06testimages.htm repository (accessed on 25 May 2026).
